# EQUITABLE AND SOCIALLY JUST AGE-FRIENDLY COMMUNITIES FOR OLDER LGBTQ+ ADULTS OF COLOR

**DOI:** 10.1093/geroni/igae098.2138

**Published:** 2024-12-31

**Authors:** Austin Oswald, Lujira Cooper

**Affiliations:** Dalhousie University, Nova Scotia, Canada; Brooklyn Society for Ethical Culture, Brooklyn, New York, United States

## Abstract

The age-friendly movement has been critiqued for inadequately addressing the needs of historically disadvantaged populations, like older LGBTQ+ adults of color. This presentation describes a multi-year participatory action research project with five older Black lesbian women aged between 65 and 75. We hosted five focus groups where participants shared their daily experiences living in an age-friendly city and proposed ideas to more inclusively meet the needs of older LGBTQ+ adults of color within age-friendly activities. Focus group data were analyzed thematically, and four themes were identified which expand existing age-friendly frameworks, promoting greater equity and justice: 1) attending to the interaction between identities and structures throughout the life course, 2) understanding the impact of historical and contemporary experiences of violence and discrimination on health and aging, 3) addressing the evolving sociopolitical landscape regarding the human rights and freedoms of diverse older adults, and 4) developing interventions that create safe and affirming spaces for older LGBTQ+ adults of color. While mainstream age-friendly frameworks are widely acknowledged for enhancing the health, independence, and social connectivity of older adults, addressing the unique challenges associated with aging while navigating multiple intersecting marginalized identities requires a critical perspective. The findings of this research underscore the necessity of culturally tailored age-friendly frameworks and interventions to address the distinct needs and experiences of older LGBTQ+ adults of color. We argue that gerontology must seriously consider the impact of privilege and oppression on the health and aging experiences of vulnerable and underserved populations within age-friendly communities.

